# OcclusionChip: A functional microcapillary occlusion assay complementary to ektacytometry for detection of small-fraction red blood cells with abnormal deformability

**DOI:** 10.3389/fphys.2022.954106

**Published:** 2022-08-25

**Authors:** Yuncheng Man, Ran An, Karamoja Monchamp, Zoe Sekyonda, Erdem Kucukal, Chiara Federici, William J. Wulftange, Utku Goreke, Allison Bode, Vivien A. Sheehan, Umut A. Gurkan

**Affiliations:** ^1^ Department of Mechanical and Aerospace Engineering, Case Western Reserve University, Cleveland, OH, United States; ^2^ Division of Hematology and Oncology, School of Medicine, Case Western Reserve University, Cleveland, OH, United States; ^3^ Department of Biomedical Engineering, Case Western Reserve University, Cleveland, OH, United States; ^4^ Aflac Cancer & Blood Disorders Center Children’s Healthcare of Atlanta, Emory University School of Medicine, Atlanta, GA, United States; ^5^ Case Comprehensive Cancer Center, Case Western Reserve University, Cleveland, OH, United States

**Keywords:** red blood cell deformability, ektacytometry, sickle cell disease, occlusion, hypoxia, biomarkers

## Abstract

Red blood cell (RBC) deformability is a valuable hemorheological biomarker that can be used to assess the clinical status and response to therapy of individuals with sickle cell disease (SCD). RBC deformability has been measured by ektacytometry for decades, which uses shear or osmolar stress. However, ektacytometry is a population based measurement that does not detect small-fractions of abnormal RBCs. A single cell-based, functional RBC deformability assay would complement ektacytometry and provide additional information. Here, we tested the relative merits of the OcclusionChip, which measures RBC deformability by microcapillary occlusion, and ektacytometry. We tested samples containing glutaraldehyde-stiffened RBCs for up to 1% volume fraction; ektacytometry detected no significant change in Elongation Index (EI), while the OcclusionChip showed significant differences in Occlusion Index (OI). OcclusionChip detected a significant increase in OI in RBCs from an individual with sickle cell trait (SCT) and from a subject with SCD who received allogeneic hematopoietic stem cell transplant (HSCT), as the sample was taken from normoxic (pO2:159 mmHg) to physiologic hypoxic (pO2:45 mmHg) conditions. Oxygen gradient ektacytometry detected no difference in EI for SCT or HSCT. These results suggest that the single cell-based OcclusionChip enables detection of sickle hemoglobin (HbS)-related RBC abnormalities in SCT and SCD, particularly when the HbS level is low. We conclude that the OcclusionChip is complementary to the population based ektacytometry assays, and providing additional sensitivity and capacity to detect modest abnormalities in red cell function or small populations of abnormal red cells.

## Introduction

The red blood cell (RBC), or erythrocyte, is the most abundant blood cell type in the human body. RBCs are biconcave-shaped and anucleated, with a diameter of 8 μm and a thickness of 2 μm (Mohandas and Gallagher, 2008; Myers and Lam, 2021). In healthy individuals, RBCs are easily deformable, and flexibly pass through narrow blood vessels smaller than 8 μm to accomplish oxygen delivery in the microvasculature (Skalak and Branemark, 1969; Qiu et al., 2018). However, RBC deformability can be significantly compromised in certain conditions, such as in sickle cell disease (SCD). SCD is the most common inherited blood disorder. It is an autosomal recessive disease, affecting approximately 100,000 people in the US and millions of people worldwide (Alapan et al., 2014; Campbell et al., 2020). It is caused by the replacement of the amino acid glutamic acid with the less polar amino acid valine at the sixth position of the beta chain, leading to the production of sickle hemoglobin (HbS) (Barabino et al., 2010). HbS molecules adhere to one another, forming long, stiff polymer chains in RBCs, distorting the red cell into the characteristic sickle shape and decreasing the RBC deformability (Wood et al., 2012; Li et al., 2017; Qiang et al., 2021). In SCD, sickle RBCs are rapidly destroyed in the microcirculation, causing chronic anemia and vascular inflammation. Longstanding hemolysis and free heme damages the endothelium, which ultimately leads to widespread organ damage, lifelong morbidity, and early mortality (Platt et al., 1994; Rees et al., 2010; Manwani and Frenette, 2013; Zhang et al., 2016; Kucukal et al., 2020; An et al., 2021; Man et al., 2021b; Lu et al., 2021). With recent advancements in the understanding of SCD pathophysiology, and implementation of therapeutic treatments such as blood transfusion, antibiotics, and disease modifying agents (e.g., hydroxyurea (HU), voxelotor, crizanlizumab), individuals with SCD now have improved quality of life (Davies and Gilmore, 2003; Morrone et al., 2018; Carden and Little, 2019; Blair, 2020a; Man et al., 2020a; Blair, 2020b; Noomuna et al., 2020; Glaros et al., 2021; Manwani, 2021). However, these novel therapeutic agents are often expensive, and we lack a standardized biomarker assay for patient screening and dose response monitoring needed for optimal use. A validated, standardized biomarker assay of red cell function could be used to improve patient care through allowing clinicians to implement a targeted, personalized treatment strategy based on the individual’s red cell function.

SCD is a clinically heterogeneous disease, and many clinical parameters have been extensively studied to correlate with disease status, such as %fetal hemoglobin, white blood cell count, and reticulocyte count (Brittenham et al., 1985; Litos et al., 2007; Du et al., 2015; Alapan et al., 2016b; Carden et al., 2020). RBC deformability is another key biomarker for assessing disease severity in SCD (Shevkoplyas et al., 2006; Alapan et al., 2016a; Ribeil et al., 2017; Catarino et al., 2019; Lu et al., 2020; Gurkan, 2021; Inanc et al., 2021). In the current laboratory and clinical settings, RBC deformability is typically measured by ektacytometry. A common type of ektacytometry used in translational and clinical research dilutes RBC to a low hematocrit level and adds them to a highly viscous medium. The sample is loaded in between a stationary cylinder and a rotatory cup (Groner et al., 1980; Da Costa et al., 2016; Renoux et al., 2016). As a result, the RBCs deform under shear flow conditions, and their laser diffraction patterns are captured during their passage between the laser and the camera. At the end of the measurement, Elongation Index (EI), which denotes the relative lengths of the major and minor axes of the RBC deformation pattern, is reported as a dimensionless metric of deformability at a known shear stress value (Parrow et al., 2018; Piety et al., 2021). However, it is useful to note that the measured EI represents the average deformability of RBCs in bulk. Therefore, it has been recognized that ektacytometry is incapable of probing small-volume abnormal RBCs within the entire cell population. Such limitation is potentially life-threatening to people with SCD, since insufficient measurements of RBC subpopulations may lead to false determination of disease status and consequently inappropriate treatment strategy. A single cell-based assay with finer resolution would complement to ektacytometry and significantly benefit a more accurate RBC deformability measurement.

We have previously developed the OcclusionChip technology (Man et al., 2020b; Man et al., 2021a), which mimics the capillary bed architecture so it can measure the ability of RBCs to navigate through narrow microcapillaries. We have demonstrated the utility of the OcclusionChip in a number of pathological scenarios, including SCD, hemoglobin SC disease, malaria, renal failure, hereditary spherocytosis, and blood storage lesions. There are two goals in this study: to determine the reproducibility of values generated by the standardized OcclusionChip assay and a commercially available ektacytometry; and to determine if the OcclusionChip assay could complement to ektacytometry for detection of abnormal RBCs in small fractions, which ektacytometry is incapable of. To test the relative merits of the two devices, we measured the EI and OI of blood samples containing gradient concentrations of glutaraldehyde-stiffened RBCs. We also tested clinical blood samples obtained from subjects with SCD using oxygen gradient ektacytometry, which measures the EI at a range of pO_2_ level (159∼5 mmHg), and the hypoxic OcclusionChip assay, which measures OI at physiologic hypoxic pO_2_ level (45 mmHg). We found that the OcclusionChip assay is more sensitive to alterations in RBC deformability in SCD, particularly when %sickle hemoglobin is small, thus able to complement to ektacytometry for more accurate assessment of patient clinical status.

## Materials and methods

### OcclusionChip device design and fabrication

**FIGURE 1:**
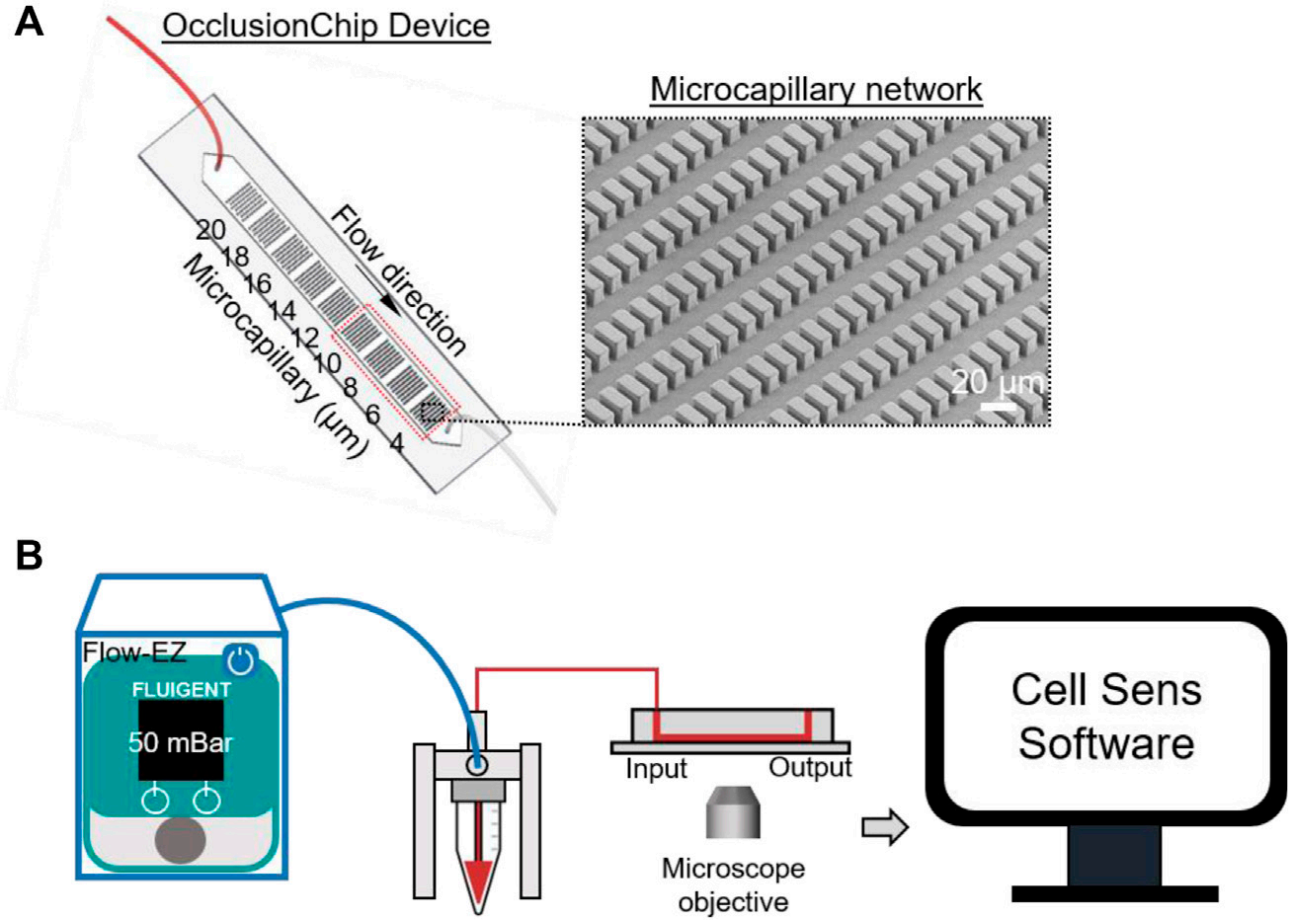
Schematic illustration of the OcclusionChip device used to evaluate deformability of RBCs. **(A)** The OcclusionChip device consists of microcapillary networks from 20 μm down to 4 μm along the flow direction for retention of RBCs with abnormal deformability. Inset: A scanning electron microscopy image of the 4-μm microcapillary network. **(B)** Experimental setup. RBC suspension at 20% hematocrit was pushed through the OcclusionChip microchannel at 50 mBar constant inlet pressure for 20 min using the Flow-EZ microfluidic flow control unit. The OcclusionChip device is mounted on the stage of an inverted microscope and imaged using the Cell Sens software.

Figure 1A shows the layout of the OcclusionChip device. Briefly, a gradient of capillary network-inspired micropillar arrays were embedded into the microfluidic channel forming microcapillaries from 20 μm down to 4 μm. Such design mimics the non-uniform, continuously changing capillaries in the capillary bed, allowing RBCs with significantly decreased deformability retained at in the upstream array with coarser openings, and those with modestly decreased deformability retained in the downstream array with finer openings. Moreover, the micropillar arrays were coupled with two 60-μm side passageways mimicking the arteriovenous anastomoses in the capillary bed (Figure 1A). This feature helps regulate blood flow such that when upstream portion of the array is completely obstructed, incoming RBCs can flow into the microfluidic anastomosis, and re-enter the array at the downstream portion.

The fabrication process of the OcclusionChip device started from spin-coating a uniform SU-8 2010 (Thermo Fisher Scientific, Waltham, MA, United States) layer at 2,500 rpm on a 3-inch silicon wafer (University Wafers, Boston, MA, United States). Following 4-min soft bake at 95°C, the wafer was UV-patterned under a photomask (Compugraphics, Austin, TX) and was post-exposure baked for 4 min at 95°C. The wafer was then developed in propylene glycol methyl ether acetate (Sigma Aldrich, St. Louis, MO, United States), and kept at 110°C overnight for hard bake. Thereafter, the wafer was surface passivated with trichloroĲ1H,1H,2H,2H-perfluorooctyl) saline (Sigma Aldrich) for 2 h under vacuum. Polydimethylsiloxane (PDMS) mixed with curing agent (10:1 v/v ratio) was degassed and casted on top of the wafer. Following incubation at 80°C overnight, the PDMS replicas were punched with inlet and outlet ports and were bonded on standard microscope slides (Electron Microscopy Sciences, Hatfield, PA, United States) through oxygen plasma treatment. Fidelity of the micropillar arrays following the fabrication process was confirmed via scanning electron microscopy (SEM) imaging (Figure 1A inset). Following tubing assembly (0.010″ ID × 0.030″ OD, Cole-Parmer, Vernon Hills, IL, United States), the OcclusionChip microchannel was rinsed with ethanol and phosphate-buffered saline (PBS, 1X), blocked with 2% bovine serum albumin (BSA, ProSpec-Tany TechnoGene Ltd., East Brunswick, NJ), and stored at 4°C overnight.

### Blood sample acquisition and OcclusionChip assay operation

De-identified venous blood samples were drawn into EDTA-containing vacutainers at University Hospitals Cleveland Medical Center (UHCMC) in Cleveland, Ohio under the Institutional Review Board approved protocol (IRB 05-14-07C), and stored at 4°C before being tested. All assays were completed within 24 h of venipuncture. Informed consent was obtained from all study participants. Hematologic parameters of the subjects with SCD were extracted from the medical records. Leukocyte-reduced whole blood samples (Pall Corporation, Port Washington, NY, United States) were placed in Eppendorf tubes and centrifuged at 500 g, after which the plasma and near-plasma portion of the RBC layer were carefully discarded. The remaining RBCs were washed twice with PBS, and 80 μl of RBCs were taken from the bottom of the tube and were re-suspended in PBS at 20% hematocrit (400 μl total volume). The OcclusionChip works with a digital pump (Flow-EZ^TM^, Fluigent, Lowell, MA, United States) for precise microfluidic flow control, and an Olympus IX83 inverted motorized microscope with Cell Sens software for high-resolution imaging (Figure 1B). Before sample loading, the OcclusionChip microchannel was rinsed with PBS. The RBC sample was thereafter loaded and allowed to perfuse at 50 mBar for 20 min. The microchannel was then washed with PBS for 20 min and imaged. Microscope images were processed by Adobe Photoshop software (San Jose, CA, United States). Each data point was generated by a fresh OcclusionChip device. The OcclusionChip device was discarded following single time use to prevent sample-to-sample contamination.

### Hypoxic OcclusionChip assay

To create a physiologic hypoxic flow condition in the OcclusionChip assay, we previously described a micro-gas exchanger to couple the OcclusionChip device (Kim et al., 2017). Briefly, a gas permeable inner tubing (0.012″ ID × 0.025″ OD, Cole-Parmer) was placed within a gas impermeable outer tubing (0.0625″ ID × 0.125″ OD, Cole-Parmer), and the 5% CO_2_ and 95% N_2_ controlled gas was allowed in the spacing. Such design enables the blood flow in the inner tubing to exchange oxygen through the tubing wall. Our previous simulation results show that the micro-gas exchanger leads to SpO_2_ of 83% (which approximately translates to pO_2_ of 45 mmHg) in the blood flow when it reaches the microchannel. A small chamber built with polymethyl methacrylate (PMMA) and double-sided adhesive (DSA), was used to seal the PDMS microchannel with the controlled gas. In the hypoxic OcclusionChip assay, the RBC sample was allowed to perfuse for 2 min, instead of 20 min. The rest experimental condition is the same as stated above.

### Occlusion index generalization

We have previously reported the generalization of an intuitive standardized parameter, Occlusion Index (OI), which represents the overall occlusion percentage of a specific artificial capillary network (Supplementary Figure S1). The area of interest in the OcclusionChip is defined as the 4-μm to 10-μm micropillar arrays (Figure 1A, red dash rectangle), since typical capillary dimension is within 5–10 μm, and no appreciable microcapillary occlusion was observed in the other arrays with larger openings.

### Ektacytometry measurements and elongation index

Ektacytometry (Lorrca, RR Mechatronics, Zwaag, Netherlands) measurements were performed according to the manufacturers’ specifications. For the deformation assay, 25 μl of whole blood samples were diluted in 5 ml of Elon-ISO solution included in the LORRCA reagents, and 800 μl of diluted blood samples were injected into the instrument and analyzed at shear stress ranging from 0.3 to 30.0 Pa. For oxygen gradient ektacytometry, 50 µl of whole blood samples were diluted in 5 ml of Oxy-ISO solution, and approximately 1.6 ml of the diluted blood samples were injected into the instrument and analyzed at the shear stress of 30 Pa under pO_2_ from 159 mmHg down to ∼5 mmHg. Elongation Index (EI) was calculated as EI = (A − B)/(A + B), where A and B represent the major and minor axes of the deformed RBC diffraction pattern. EI results are reported at three shear stress levels, 3 Pa (low), 16.87 Pa (medium), and 30 Pa (high) for the deformation assay, and at 30 Pa at the normoxic pO_2_ level (159 mmHg) and the physiologic hypoxic pO_2_ level (45 mmHg) for oxygen gradient ektacytometry in this study.

### Statistical analysis

Data are reported as mean ± standard deviation (SD). Statistical analyses were carried out using Minitab 20 Software (Minitab Inc, State College, PA, United States). Data were initially analyzed for normality, which was followed by appropriate two-group comparison tests: paired *t*-test for paired groups, student’s t-test for unpaired, normally distributed groups, and non-parametric Mann-Whitney U test for non-normal groups. Statistical significance was defined with *p*-value < 0.05 (*p* < 0.05).

## Results

### Two-user validation of ektacytometry and the OcclusionChip

**FIGURE 2:**
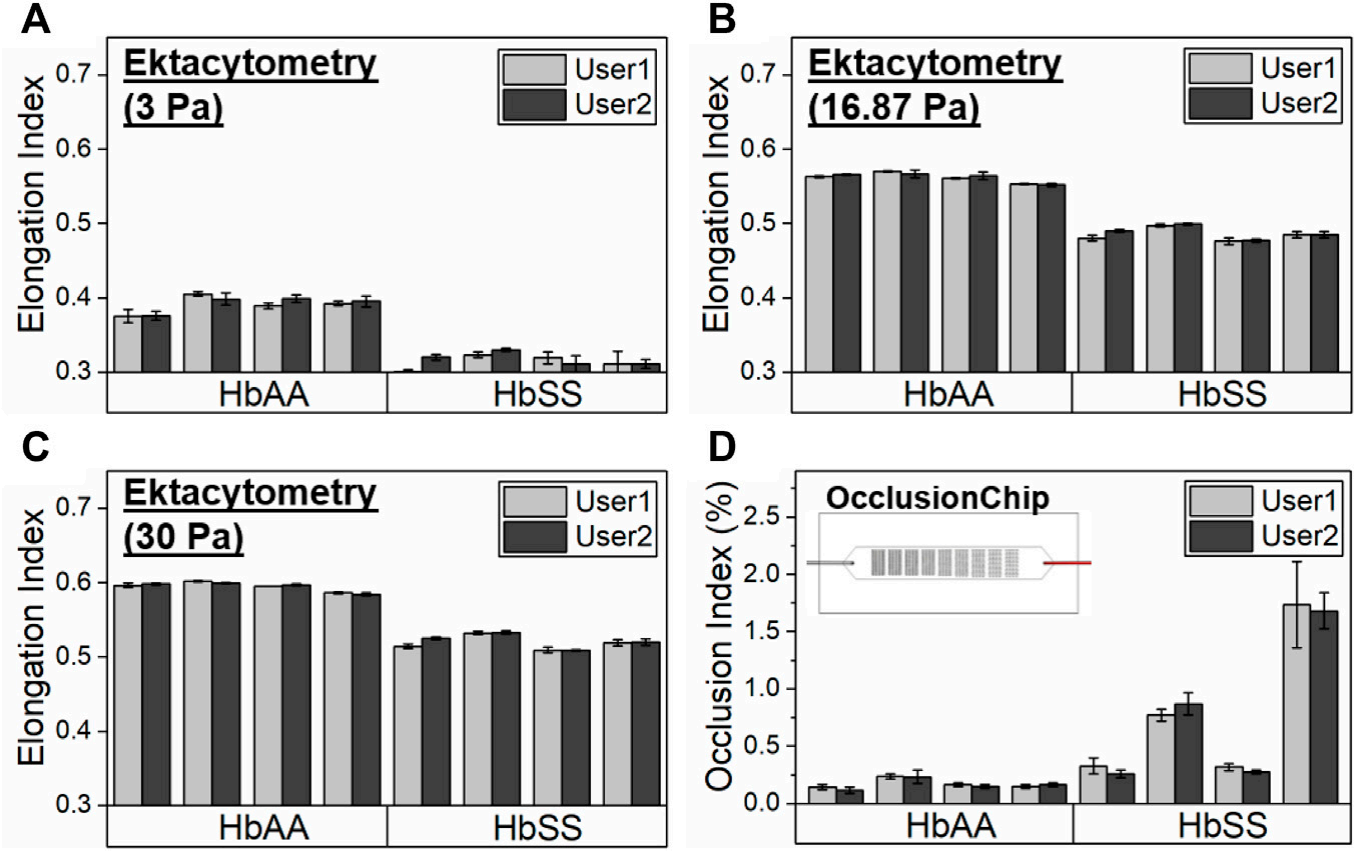
Validation of the process reproducibility of ektacytometry and the OcclusionChip by two users. Elongation Index (EI) results by ektacytometry of 4 healthy donors (HbAA) and 4 subjects with homozygous SCD (HbSS) at three shear stress levels, **(A)** 3 Pa, **(B)** 16.87 Pa, and **(C)** 30 Pa. **(D)** Occlusion Index (OI) results by the OcclusionChip of 4 healthy donors (HbAA) and 4 subjects with homozygous SCD (HbSS). Values shown are mean ± standard deviation (N = 3).

We validated ektacytometry and the OcclusionChip with two different users; 3 repeat runs were performed by each user, using blood samples from 4 HbAA and 4 HbSS subjects. The hematological parameters of the HbSS subjects are shown in Table 1. The EI of the HbAA subjects ranged from 0.365 to 0.407, 0.550–0.571, and 0.582–0.603 at the shear stress level of 3 Pa, 16.87 Pa, and 30 Pa, respectively (Figures 2A–C). The EI of the HbSS subjects ranged from 0.292 to 0.332, 0.472–0.500 and 0.505–0.534 at the same shear stress levels (Figures 2A–C). The range of OI of HbAA and HbSS subjects were 0.09%–0.24% and 0.23%–2.06%, respectively (Figure 2D). To analyze the process reproducibility, we quantified Coefficient of Variation (CV) (= SD/mean * 100%) which is summarized in Table 2. 20% was used as the threshold for the validation according to US Food and Drug Administration (FDA) Bioanalytical Method Validation (≥ three replicates in at least six runs) (U. S. Food, and Drug Administration, 2018). We found the range of single-user CV among the tested HbAA and HbSS samples in ektacytometry as 0.63%–5.35%, 0.65%–5.35%, and 0.00%–0.71%, with an average of 1.77%, 0.59%, and 0.36% at the shear stress level of 3 Pa, 16.87 Pa, and 30 Pa, respectively. On the other hand, we found the range of single-user CV among the tested HbAA and HbSS samples in the OcclusionChip assay as 6.44%–24.63%, with an average of 14.71%. These results show that while both technologies are reproducible, with two-user average CV less than 20%, ektacytometry is much more consistent and reproducible than the OcclusionChip.

**TABLE 1:** Subject hematological parameters for process validation. Values shown are mean ± standard deviation (range). *N* = 4 in each group.

Clinical variables	OcclusionChip	Ektacytometry	Normal range
	Mean ± SD (range)	Mean ± SD (range)	
Age (years)	44 ± 6 (38–51)	34 ± 8 (22–41)	N/A
Hemoglobin (g/dl)	8.2 ± 1.7 (6.4–9.9)	8.4 ± 0.9 (7.6–9.6)	12.0–18.0
Red blood cell count (10^12^/L)	3.4 ± 1.4 (2.0–4.8)	2.5 ± 0.2 (2.2–2.8)	4.2–6.1
Hematocrit (%)	25.7 ± 7.0 (19.1–31.9)	24.9 ± 2.4 (21.8–27.6)	36.0–50.0
Absolute reticulocyte count (10^9^/L)	370 ± 175 (184–590)	331 ± 228 (189–671)	20–150
Lactate dehydrogenase (U/L)	420 ± 318 (134–745)	298 ± 33 (256–336)	140–280
Ferritin (μg/L)	1709 ± 2,484 (15–5,280)	2,615 ± 2,206 (216–5,520)	12–300
White blood cell count (10^9^/L)	10.2 ± 2.6 (7.7–12.5)	12.0 ± 4.1 (7.9–17.2)	4.0–11.0
Absolute neutrophil count (10^6^/L)	6,290 ± 2,705 (2,680–8,880)	6,048 ± 2,145 (3,130–8,300)	1,500–8,000
Platelet count (10^9^/L)	400 ± 132 (268–546)	475 ± 128 (326–637)	150–400
Hemoglobin S (%)	51.5 ± 9.0 (41.3–62.4)	61.7 ± 28.0 (20.5–79.9)	N/A
Hemoglobin A (%)	35.4 ± 6.3 (27.9–41.4)	21.1 ± 25.8 (1.9–57.1)	99–100
Hemoglobin F (%)	1.9 ± 1.9 (0.5–4.6)	7.9 ± 7.5 (1.1–16.5)	0–0.9

**TABLE 2:** Coefficient of variation (CV = standard deviation/mean) of the OcclusionChip and ektacytometry validation results. Values shown are in percentage.

CV (%)	OcclusionChip		Ektacytometry					
			3Pa		16.87 Pa		30 Pa	
	User1	User2	User1	User2	User1	User2	User1	User2
HbAA	15.82	23.84	2.33	1.56	0.20	0.10	0.51	0.19
	9.2	24.63	0.65	1.88	0.10	0.80	0.17	0.17
	11.08	12.34	0.90	1.18	0.21	0.82	0.00	0.39
	9.81	10.79	0.82	1.86	0.18	0.31	0.20	0.36
HbSS	21.67	12.56	0.69	1.26	0.73	0.31	0.59	0.38
	6.84	11.14	1.12	0.63	0.31	0.23	0.29	0.29
	9.62	6.44	2.45	3.70	0.99	0.44	0.71	0.11
	21.66	9.52	5.35	1.96	0.86	0.83	0.69	0.68
Average	14.71		1.77		0.59		0.36	

### Both ektacytometry and the OcclusionChip enable detection of abnormal red blood cells deformability under normoxia in sickle cell disease

**FIGURE 3:**
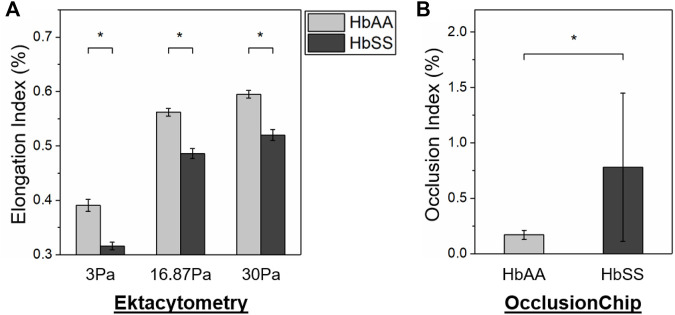
Both ektacytometry and the OcclusionChip can effectively detect the abnormal RBC deformability in SCD. **(A)** At all the three shear stress levels (3, 16.87 and 30 Pa), the Elongation Index (EI) of RBCs from subjects with homozygous SCD (HbSS) is significantly lower than those from healthy donors (HbAA) by ektacytometry (*p* < 0.05, student’s t-test). **(B)** The Occlusion Index (OI) of RBCs from subjects with homozygous SCD (HbSS) is significantly greater than those from healthy donors (HbAA) by the OcclusionChip assay (*p* < 0.05, Mann-Whitney). Values shown are mean ± standard deviation (N = 4). * denotes a statistical significance between HbAA and HbSS (*p* < 0.05).

We next compared the ability of ektacytometry and the OcclusionChip to evaluate abnormal RBC deformability in SCD. We found that in ektacytometry, the EI of the HbSS subjects is significantly lower than that of the HbAA subjects at all the reported shear stress levels (Figure 3A, 0.316 ± 0.007 vs. 0.391 ± 0.011 at 3 Pa, 0.486 ± 0.009 vs. 0.562 ± 0.007 at 16.87 Pa, and 0.520 ± 0.010 vs. 0.595 ± 0.007 at 30 Pa for HbSS vs. HbAA, *p* < 0.05, student’s *t*-test). Similarly, in the OcclusionChip assay we found that the OI of the HbSS subjects is significantly greater than that of the HbAA subjects (Figure 3B, 0.78 ± 0.67 vs. 0.17 ± 0.04 for HbSS vs. HbAA, *p* < 0.05, Mann-Whitney). These results confirmed that both ektacytometry and the OcclusionChip can effectively measure the abnormal deformability in SCD under oxygenated conditions by yielding values that are significantly different between individuals with SCD and healthy individuals.

### OcclusionChip enables detection of glutaraldehyde-stiffened red blood cells in small fractions

**FIGURE 4:**
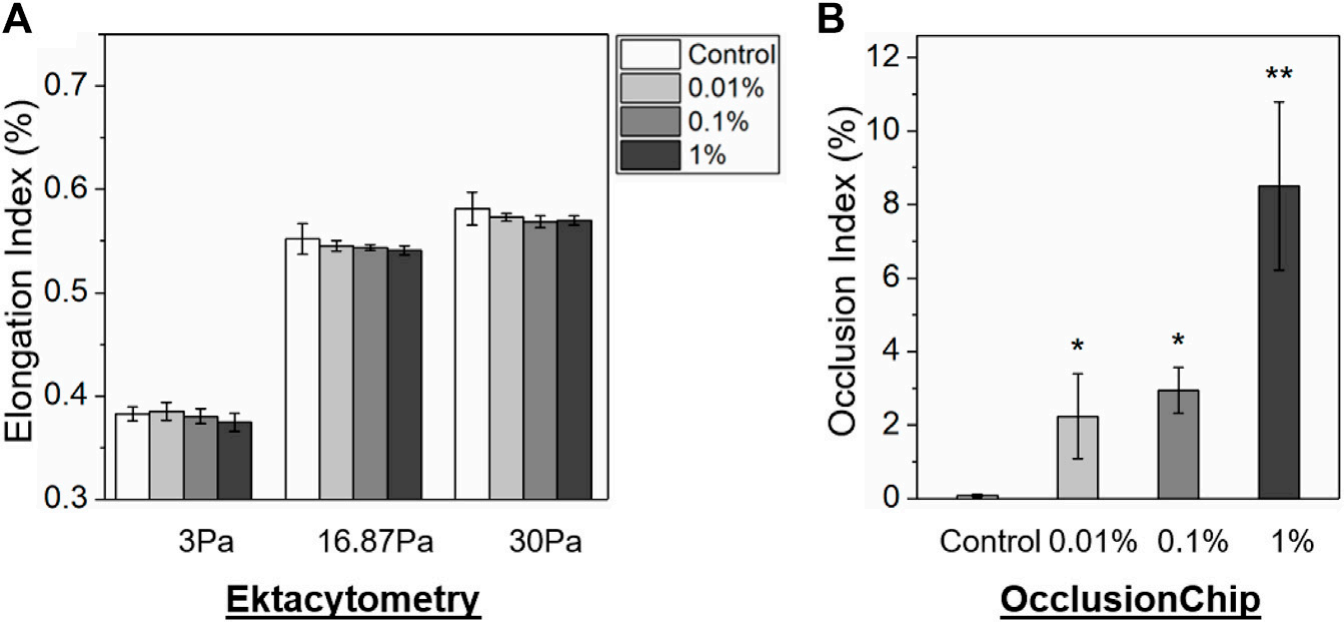
The OcclusionChip enables detection of small-fraction glutaraldehyde-stiffened RBCs in mixtures with normal RBCs. **(A)** Elongation Index (EI) results of glutaraldehyde-stiffened RBC and normal RBC mixtures at four different ratios, 0% (as control), 0.01%, 0.1%, and 1%, at three shear stress levels (3, 16.87 and 30 Pa) by ektacytometry. Although EI decreased as the concentration of glutaraldehyde-stiffened RBCs increased, no statistical significance was found between mixtures with different ratios (*p* > 0.05). **(B)** Occlusion Index (OI) results on the same RBC mixtures. OI of RBC mixtures containing 0.01% or 0.1% glutaraldehyde-stiffened RBCs is significantly greater than the control, and OI of RBC mixtures containing 1% glutaraldehyde-stiffened RBCs is significantly greater than other groups. Values shown are mean ± standard deviation (N = 3). * denotes a statistical significance compared with control (*p* < 0.05, paired *t*-test), and ** denotes a statistical significance compared with other groups (*p* < 0.05, paired t-test).

We then simultaneously carried out ektacytometry measurement and the OcclusionChip assay using samples containing abnormal RBCs in small fractions. To generate these samples, glutaraldehyde-stiffened RBCs (20% hematocrit in 0.08% glutaraldehyde in PBS, 10-min incubation at room temperature) were mixed with normal RBCs at the volume concentrations of 0% (as control), 0.01%, 0.1%, and 1%. The mixed samples were thereafter tested, with 3 repeats on each samples. We found that even though EI decreased as the concentration of glutaraldehyde-stiffened RBCs, no significant difference was observed in EI between those samples (Figure 4A, 0.383 ± 0.007 vs. 0.385 ± 0.009 vs. 0.380 ± 0.007 vs. 0.374 ± 0.009 at 3 Pa, 0.552 ± 0.015 vs. 0.545 ± 0.005 vs. 0.544 ± 0.003 vs. 0.541 ± 0.005 at 16.87 Pa, and 0.581 ± 0.016 vs. 0.573 ± 0.004 vs. 0.569 ± 0.006 vs. 0.570 ± 0.005 at 30 Pa for 0% (control) vs. 0.01% vs. 0.1% vs. 1% glutaraldehyde-stiffened RBCs, *p* > 0.05). We found that OI increased as the concentration of glutaraldehyde-stiffened RBCs increased in the OcclusionChip (Figure 4B, 0.08 ± 0.03% vs. 2.24 ± 1.16% vs. 2.94 ± 0.62% vs. 8.51 ± 2.29% for 0% (control) vs. 0.01% vs. 0.1% vs. 1% glutaraldehyde-stiffened RBCs). Moreover, we found significantly greater OI in samples with 0.01% or 0.1% glutaraldehyde-stiffened RBCs than the control, and the samples with 1% glutaraldehyde-stiffened RBCs had the highest OI (Figure 4B, *p* < 0.05, paired t-test). These results suggest that the OcclusionChip enables detection of abnormal RBCs in small fractions, which ektacytometry does not.

### OcclusionChip enables detection of hypoxia-decreased red blood cells deformability in sickle cell trait and sickle cell disease, when %sickle hemoglobin is low

**FIGURE 5:**
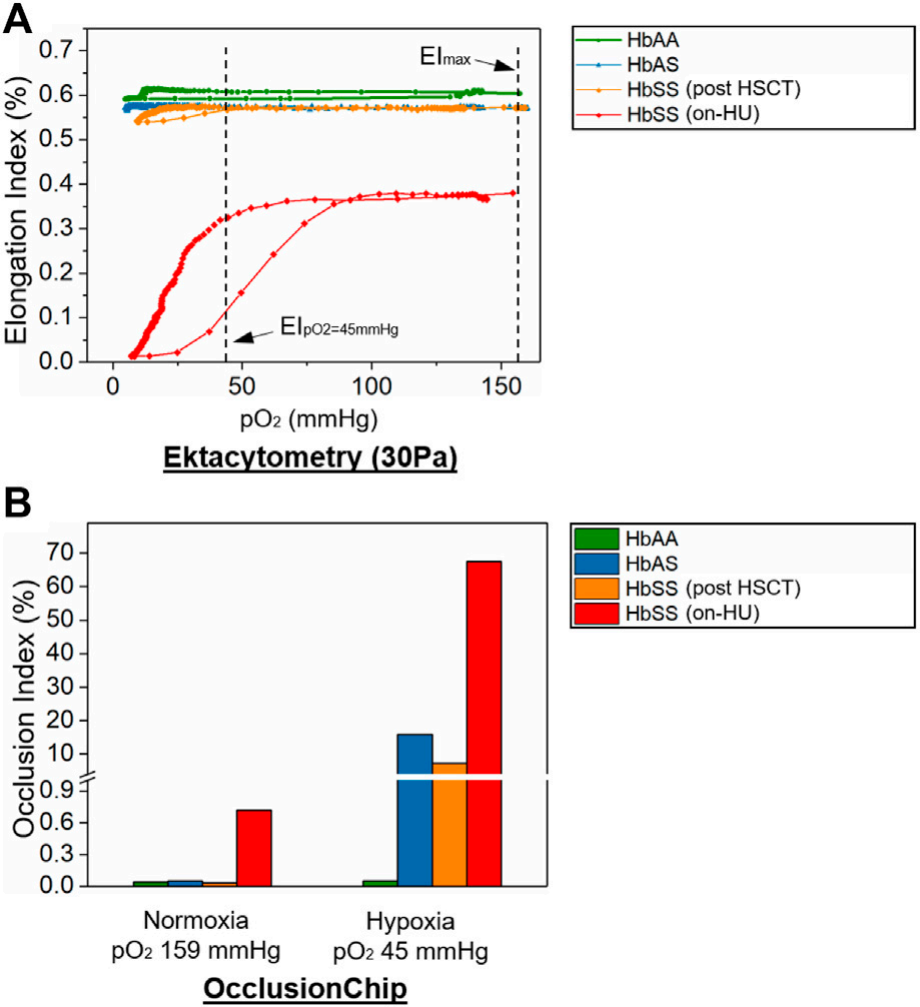
OcclusionChip enables detection of HbS-related RBC abnormalities under hypoxia in SCT and SCD when HbS level is low. Shown are **(A)** Elongation Index (EI) results by oxygen gradient ektacytometry and **(B)** Occlusion Index (OI) results by the OcclusionChip on clinical blood samples acquired from a healthy donor (HbAA), a subject with SCT (HbAS), a subject with homozygous SCD (HbSS) post allogeneic hematopoietic stem cell transplant (HSCT), and a subject with homozygous SCD (HbSS) who were on-hydroxyurea (HU), under normoxic (159 mmHg) and physiologic hypoxic pO_2_ level (45 mmHg). EI decreased from 0.380 to 0.325 in the sample from the on-HU HbSS subject, and remained similar in the samples from the HbAS subject and the HbSS subject who received HSCT under hypoxia. However, OI increased from 0.05% to 15.89%, from 0.03% to 7.23%, and from 0.72% to 67.52% in the samples from the HbAS subject, the HbSS subject post HSCT, and the on-HU HbSS subject, respectively, under hypoxia.

We further analyzed clinical blood samples from a healthy donor (HbAA), a subject with SCT (HbAS), and a subject with homozygous SCD on HU (HbSS) using oxygen gradient ektacytometry and the normoxic/hypoxic OcclusionChip assay. Under normoxia, both assays showed similar results when comparing the samples from the subjects with HbAA or HbAS (EI_max_: 0.604 vs. 0.569 and OI: 0.04% vs. 0.05% for HbAA vs. HbAS), and a significantly different result in the sample from the subject with HbSS on-HU (EI_max_: 0.380 and OI: 0.72% for HbSS) (Figure 5). However, oxygen gradient ektacytometry showed no difference in EI under physiologic hypoxia (Blaisdell et al., 2000) compared to normoxia in the sample from the subject with HbAS (Figure 5A, 0.573 vs. 0.569 for EI_pO2=45mmHg_ vs. EI_max_). Meanwhile, the OcclusionChip assay showed a significantly increased OI under physiologic hypoxia compared to normoxia (Figure 5B, OI = 15.89% vs. 0.05% for hypoxia vs. normoxia). These results suggest that the OcclusionChip enables detection of HbS-related RBC abnormalities under hypoxia for SCT identification, which oxygen gradient ektacytometry does not. Notably, both assays were able to detect the significantly decreased RBC deformability under hypoxia in the sample from the subject with HbSS SCD compared to normoxia (0.325 vs. 0.380 for EI_pO2=45mmHg_ vs. EI_max_ and 67.52 vs. 0.72 for OI hypoxia vs. normoxia) (Figure 5).

We further tested a clinical sample from a subject with HbSS SCD who received allogeneic hematopoietic stem cell transplant (HSCT), a curative therapy for SCD (donor genotype: HbAS, Supplementary Table S1) using the two assays. Of note, donor chimerism 102 days following the treatment was >99%, and our measurements were performed 117 days following the treatment. Under normoxia, both assays showed results similar to what was observed in samples from subjects with HbAA or HbAS (EI_max_: 0.572 and OI: 0.03%). However, under physiologic hypoxia, oxygen gradient ektacytometry showed no difference in EI compared to normoxia in the sample (Figure 5A, 0.572 vs. 0.572 for EI_pO2=45mmHg_ vs. EI_max_), but the OcclusionChip assay showed a significantly increased OI was observed compared to normoxia (Figure 5B, OI = 7.25% vs. 0.03% for hypoxia vs. normoxia). These results further confirmed that the OcclusionChip enables detection of HbS-related RBC abnormalities in SCD, particularly when the HbS level is low, which ektacytometry does not, thus complementing to ektacytometry for more accurate assessment of patient clinical status.

## Discussion

In this study, we analytically validated the OcclusionChip for assessment of microcapillary occlusion mediated by RBC deformability, and compared it to commercially available ektacytometry. Our two-user validation results established that the OcclusionChip assay results are reproducible, with an average CV (= SD/mean * 100%) of 14.71%, below the threshold (20%) set by FDA (U. S. Food, and Drug Administration, 2018). Compared to our previously described version of the OcclusionChip (Man et al., 2020b), we replaced the manual pump with a digital microfluidic pump and introduced a leukocyte-reduction procedure to improve standardization. However, compared with ektacytometry, which yielded an average CV of 0.91% in the two-user validation, the OcclusionChip assay still has a relatively higher degree of variation. We postulate that the variation can be attributed to three factors: the nature of single-cell analyses which captures greater biological variability, the device fabrication process, and the hands-on user involvement. Since the OcclusionChip assay is a single cell-based assay, it enables detection of even the subtle difference between different aliquots of the same biological sample, which is reflected in the variation in the OI. Moreover, in its current form, the OcclusionChip device fabrication requires a multistep cleanroom work and experienced personnel, which may induce batch-to-batch variation. Notably, hot embossing is a versatile fabrication method which uses high pressure and temperature to transfer the structures from the master into the polymer (Worgull, 2009). Its primary advantage for microfluidic applications is the ability to achieve massive, clean, and precise micro structures quickly and cost-effectively in materials that often cannot be machined utilizing other technologies. Streamlining the manufacturing of OcclusionChip design using hot embossing would significantly benefit a high-throughput testing capability. On the other hand, the current OcclusionChip assay operation still requires more user input (e.g., tubing assembly, flow control, microchannel imaging), compared to the fully automated ektacytometry measurement (Rab et al., 2021). An automated system for subsequent flow pumping and image capture and analysis would likely reduce the variation to minimum.

Our results show that both ektacytometry and OcclusionChip are able to differentiate RBC samples from HbSS blood samples to HbAA blood samples. Of note, we used different statistical methods for comparison between two populations (*t*-test vs Mann–Whitney), which was based on data normality. Further, we observed that results from two of the subjects with HbSS SCD are closed to results from normal control (HbAA). Such observation is due to the fact that the two subjects were on transfusion therapy and had high HbA percentage at the time point of blood sampling (~ 40% HbA). Since SCD is a clinically heterogeneous disease, patients at steady state with better treatment outcome may have significantly ameliorated RBC deformability (such as high HbA% following recent transfusion or high HbF% following hydroxyurea treatment). On the other hand, OI is an indicative index of RBC deformability, and RBC deformability could be inferred through comparison between individual samples (such as HbSS vs HbAA). A greater OI value indicates more severe blockage of the microcapillary network in the OcclusionChip (e.g., 100% OI indicates complete blockage), which translates to a greater potential of the RBCs blocking blood flow in human microvasculature. OcclusionChip is limited in providing a direct measurement of RBC deformability value (e.g., cell elongation index under specific shear stress levels). However, it mimics the human microvascular architecture and mechanically assesses RBCs passing through narrow openings under flow conditions. Ektacytometry measures RBC deformability values over a range of shear stress levels (deformation assay) or it provides measurement at a defined shear stress but varying medium osmolality (osmotic gradient ektacytometry) or oxygen concentration (oxygen gradient ektacytometry). The devices measure deformability through different strategies; one may argue that the OcculsionChip replicates *in vivo* conditions more faithfully.

Results reported in this study suggest that the OcclusionChip enables detection of small-volume RBCs with abnormal deformability, which ektacytometry does not, thus could complement ektacytometry for clinical testing. When we increased the concentrations of glutaraldehyde-stiffened RBCs in normal RBCs, EI measured by ektacytometry only decreased by maximal 2.35%, while OI measured by the OcclusionChip increased by more than 100 folds (for samples containing 1% glutaraldehyde-stiffened RBCs vs. control). The sharp contrast is due to the fact that ektacytometry measures the average property of a number of RBCs and the small fraction of abnormal RBCs could not cause a significant change of the measurement readout. However, in human microvasculature, components less than the dimension of RBC size only allow single cell to transit at a time, and one single RBC with decreased deformability may be sufficient to obstruct the blood flow and cause further microvascular complications, particularly in SCD. Therefore, it is critical to carry out single-cell assay when ektacytometry yields non-significant results in diseased samples. Since the OcclusionChip assay essentially measures individual RBC deformability to pass through microcapillaries, OI is a better characterization of RBC subpopulations and thus could complement to ektacytometry with a finer resolution. Further, results suggest that the OcclusionChip assay is capable of probing even 0.01% abnormal RBCs, and also differentiating samples with varying levels of abnormal RBCs (even though a statistical significance was not observed for samples containing 0.01% vs. 0.1% glutaraldehyde-stiffened RBCs). Future work may focus on characterizing the limit of detection and also the minimum resolution of the OcclusionChip.

A powerful method for assessment of RBC deformability as a function of oxygen tension, termed as oxygen gradient ektacytometry, has been developed recently and shown useful particularly in SCD (Sadaf et al., 2021). In a previous study, Rab et al. showed that oxygen gradient ektacytometry is able to detect the therapeutic effect of a novel drug, Voxelotor (GBT440), which could alter the oxygen affinity of hemoglobin in SCD thus preventing RBC sickling (Rab et al., 2019). In another study, Rab et al. showed the impact of the time from blood sampling to measurement, the amount of RBCs during testing, the camera gain settings, and the speed of deoxygenation on the measurements of oxygen gradient ektacytometry and therefore concluded that the standardization of oxygen gradient ektacytometry would allow different laboratories to compare RBC rheology study in SCD (Rab et al., 2020). Moreover, a recent study by Nardo-Marino et al. showed that oxygen gradient ektacytometry measurements correlate with key hematological parameters in children with SCD such as Hb levels, HbF, lactate dehydrogenase, and reticulocyte counts (Nardo-Marino et al., 2021). However, this study challenges oxygen gradient ektacytometry that it failed to detect the effect of hydroxyurea (HU) treatment and pain level in the pediatric population. A standardized, single cell-based microfluidic approach would complement oxygen gradient ektacytometry and significantly benefit a more comprehensive RBC biorheological assessment. Specifically, we have previously showed that the OcclusionChip enables patient stratification based on disease severity and identification of HU non-responsive individuals in SCD (Man et al., 2021a).

Envisioning the potential of the OcclusionChip assay as a clinical biomarker assay complementary to ektacytometry in SCD, we tested clinical samples under both normoxic and physiologic hypoxic conditions in the OcclusionChip and compared with oxygen gradient ektacytometry. In particular, we simultaneously tested clinical blood samples from an HbSS subject on HU, and another HbSS subject who received HSCT from an HbAS donor with approximately 100% chimerism, within 4 months of engraftment, using the two assays. While both ektacytometry and OcclusionChip measurements agree on the significantly decreased RBC deformability in the sample from the on-HU HbSS subject under normoxia compared to an HbAA sample, which was further decreased under hypoxia, they are discordant on results from the sample from the HbSS subject post HSCT. EI results show that the RBC deformability of the sample from the HbSS subject post HSCT under normoxia and physiologic hypoxia was similar to HbAA. However, our OI results challenged those results, where RBCs from the HbSS subject post HSCT caused negligible microcapillary occlusion under normoxia similar to HbAA, but led to approximately 7% occlusion of the entire microcapillary network in the OcclusionChip under physiologic hypoxia. Such observation is in accordance with the medical record that within the month when the blood sample was obtained from the subject, the subject experienced 3 pain-related acute care visits, suggesting clinical severity. These results support our assertion that the OcclusionChip enables detection of HbS-related RBC abnormalities in SCD, particularly when the HbS level is low, which ektacytometry does not, thus complementing to ektacytometry for more accurate assessment of patient clinical status. More importantly, the OcclusionChip is capable of detecting the defective RBC deformability in SCT, which can be utilized to study the variability among people with SCT and identify individuals at high risk and in need of therapeutic interventions.

One interesting finding in this study is the hypoxia-enhanced microcapillary occlusion by RBCs from an HbAS subject. Again in this case, ektacytometry was not able to detect the decreased RBC deformability under hypoxia. Such observation is attributed to the fact that RBCs from HbAS subjects and RBCs from HbSS subjects possess deferential sickling profile (unpublished data). Our ongoing study using the same blood samples from the HbAS subject and the HbSS subject (without curative therapy) tested here show that only a part of HbAS RBCs started to sickle following 10-min deoxygenation, while most HbSS RBCs were already sickled within 5 to 7-min deoxygenation (Supplementary Figure S2). We postulate that in ektacytometry, the slow, partial RBC sickling did not induce significant change of the EI of the entire cell population, but in the OcclusionChip, individual sickled RBCs were retained in the network and yield microcapillary occlusion. Our OcclusionChip results support that SCT may not be treated as a benign state (Baskurt et al., 2007). However, one limitation is that the hypoxic pO_2_ level is considered physiologic within the context of SCD, but this may not be true in SCT.

## Conclusion

In conclusion, here we present validations of the OcclusionChip and a commercially available ektacytometry, and show that the OcclusionChip enables detection of small-volume glutaraldehyde-stiffened RBCs as well as the hypoxia-decreased RBC deformability in SCT and SCD with low %sickle hemoglobin, which ektacytometry does not. These observations were attributed to the fact that the EI measured by ektacytometry is a bulk average measurement based on the laser diffraction pattern of a large number of RBCs, and OI measured by the OcclusionChip is the occlusion percentage of the microfluidic microcapillary network mediated by individual RBC deformability. Therefore, these results establish that the OcclusionChip could complement ektacytometry with a finer resolution for detection of alterations in RBC deformability in clinical settings. There are a few limitations in this study. First, additional testing such as determination of percent dense red blood cells, or distribution of HbF through determination of %F cells was not performed. Second, comparison of OcclusionChip and oxygen gradient ektacytometry was only performed on a small sample size. Third, cell count or cell volume was not considered in the experiment design. Future work needs to focus on streamlining the device fabrication and automating the flow system and the automated image analysis to reduce user input and minimize process variation. Future work will also focus on testing HbSS or HbAS samples from subjects before and after imitation of various treatments, longitudinally as they experience clinical complications, and in larger sample size to thoroughly compare the performance and clinical utility of the two technologies.

## Data Availability

The raw data supporting the conclusion of this article will be made available by the authors, without undue reservation.
