# Supplementary material for: OcclusionChip: A functional microcapillary occlusion assay complementary to ektacytometry for detection of small-fraction red blood cells with abnormal deformability
Source: Front Physiol. 2022 Aug 25;13:954106. doi: 10.3389/fphys.2022.954106 (PMC9452903; doi:10.3389/fphys.2022.954106)
Supplement: Supplementary file 1 [file DataSheet1.docx]

Supplementary Material

# Occlusion Index generalization.

Occlusion Index (OI) is defined as:

$$Occlusion Index= \frac{\sum_{i=1}^{n} {(c}_{1}+s\times(i-1))\times O_{i}}{\sum_{i=1}^{n} {(c}_{1}+s\times(i-1))\times N_{i}}\times100\%$$

Where,

$$i=The No. of the capillary network (from the outlet)$$

$$c_{1}=The smallest capillary dimension$$

$$s=Step change of capillary dimension$$

$$n=Total number of capillary networks within the area of interest$$

$$N_{i}=Total number of capillaries formed in the ith capillary network$$

$$O_{i}=Total number of capillary occlusions induced in the ith capillary network$$

We use the model shown in **Figure S1** to demonstrate the OI generalization.

# Supplementary Figures and Table

## Supplementary Figures


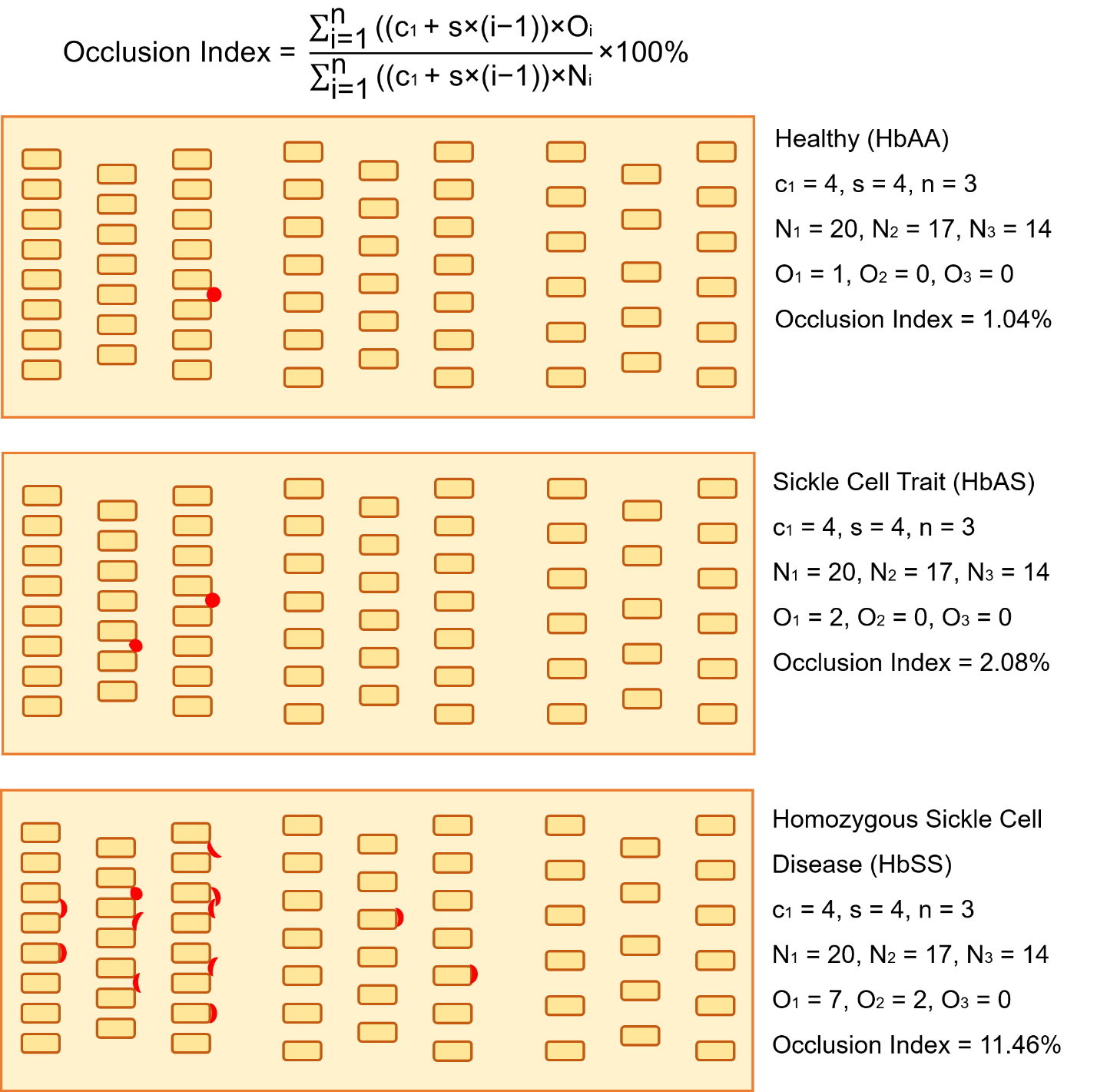


**Figure S1.** Occlusion Index is generalizable for assessing RBC-mediated microcapillary occlusion. Three identical microfluidic capillary networks are shown employing micropillar arrays assessing RBC-mediated microcapillary occlusion in three different scenarios, healthy control (HbAA), sickle cell trait (HbAS), and homozygous SCD (HbSS), are shown. Occlusion Indices for these three examples were calculated as 1.04%, 2.08%, and 11.46%, respectively.


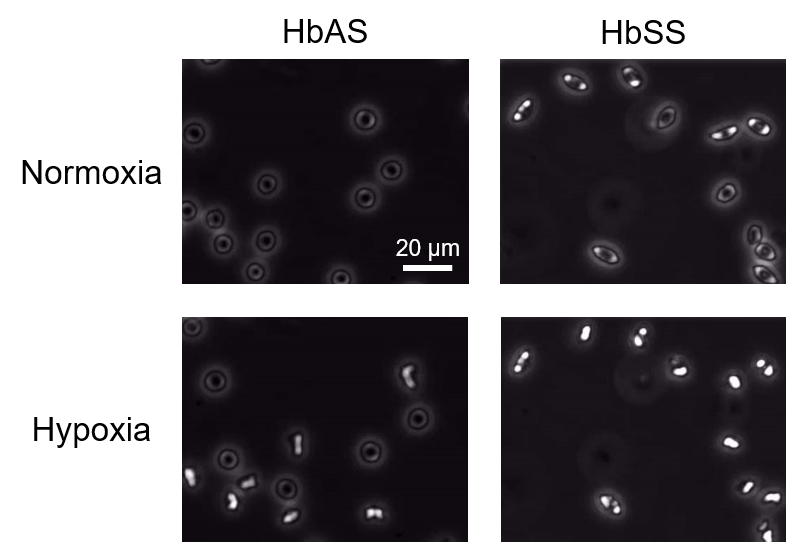


**Figure S2.** RBC sickling profile in SCT differs from SCD. RBCs from the subject with SCT (HbAS) and the subject with homozygous SCD (HbSS) on-hydroxyurea (HU) tested by oxygen gradient ektacytometry and the OcclusionChip were allowed to deoxygenate for 20 min in a PDMS-based microchannel. Representative microscopic images of showing RBC sickling of partial and entire cell population under hypoxia in the samples from the HbAS and HbSS subject, respectively.

## Supplementary Table

**Table S1. Clinical variables of the HbSS subject post HSCT and the on-HU HbSS subject.**

| Clinical variables | Normal Range  (HbAA) | The HbSS subject post HSCT | The on-HU HbSS subject |
| --- | --- | --- | --- |
| Age | N/A | 40 | 34 |
| Hemoglobin (g/dL) | 12–18 | 14.5 | 8.2 |
| MCV (fL) | 82–95 | 91 | 72 |
| WBC Count (10^9^/L) | 4–11 | 4.4 | 8.6 |
| Platelet Count (10^9^/L) | 150–400 | 165 | 452 |
| ANC (10^6^/L) | 1500–8000 | 2590 | 4750 |
| Reticulocyte (10^9^/L) | 20–150 | 34 | 258 |
| LDH (U/L) | 140–280 | 352 | 225 |
| Ferritin (μg/L) | 12–300 | 478 | 24 |
| Hemoglobin S (%) | N/A | 36.1 | 91.2 |
| Hemoglobin A (%) | 99–100 | 52.2 | 1.6 |
| Hemoglobin F (%) | 0–0.9 | 1.1 | 1.5 |
